# Supplementary material for: Testing the causal mechanism of the peninsular effect in passerine birds from South Korea
Source: PLoS One. 2021 Jan 29;16(1):e0245958. doi: 10.1371/journal.pone.0245958 (PMC7846002; doi:10.1371/journal.pone.0245958)
Supplement: S1 Table — (PDF) [file pone.0245958.s001.pdf]

Order	Family	Scientific name	Resident	Migrant
Passeriformes	Pittidae	*Pitta nympa*		O
Passeriformes	Alaudidae	*Calandrella brachydactyla*		O
Passeriformes	Alaudidae	*Alauda arvensis*	O	
Passeriformes	Hirundinidae	*Riparia riparia*		O
Passeriformes	Hirundinidae	*Hirundo rustica*		O
Passeriformes	Hirundinidae	*Hirundo daaurica*		O
Passeriformes	Hirundinidae	*Delichon urbica*		O
Passeriformes	Motacillidae	*Motacilla flava*		O
Passeriformes	Motacillidae	*Motacilla citreola*		O
Passeriformes	Motacillidae	*Motacilla cinerea*		O
Passeriformes	Motacillidae	*Motacilla grandis*	O	
Passeriformes	Motacillidae	*Dendronanthus indicus*		O
Passeriformes	Motacillidae	*Anthus rubesens*		O
Passeriformes	Motacillidae	*Anthus godlewskii*		O
Passeriformes	Motacillidae	*Ficedula narcissina*		O
Passeriformes	Motacillidae	*Motacilla alba ocularis*		O
Passeriformes	Motacillidae	*Motacilla lugens*		O
Passeriformes	Motacillidae	*Motacilla flava macronyx*		O
Passeriformes	Motacillidae	*Anthus gustavi*		O
Passeriformes	Motacillidae	*Motacilla alba baicalensis*		O
Passeriformes	Motacillidae	*Motacilla alba leucopsis*		O
Passeriformes	Motacillidae	*Anthus richardi*		O
Passeriformes	Motacillidae	*Motacilla flava simillima*		O
Passeriformes	Motacillidae	*Motacilla flava plexa*		O
Passeriformes	Motacillidae	*Anthus gustavi*		O
Passeriformes	Motacillidae	*Motacilla alba personata*		O
Passeriformes	Motacillidae	*Anthus hodgsoni*		O
Passeriformes	Campephagidae	*Pericrocotus divaricatus*		O
Passeriformes	Pycnonotidae	*Pycnonotus sinensis*		O
Passeriformes	Pycnonotidae	*Hypsipetes amaurotis*	O	
Passeriformes	Laniidae	*Lanius schach*		O
Passeriformes	Laniidae	*Lanius cristatus*		O
Passeriformes	Laniidae	*Lanius bucephalus*	O	
Passeriformes	Laniidae	*Lanius tigrinus*		O
Passeriformes	Laniidae	*Lanius sphenocercus*		O
Passeriformes	Bombycillidae	*Bombycilla japonica*		O
Passeriformes	Bombycillidae	*Bombycilla garrulus*		
Passeriformes	Troglodytidae	*Troglodytes troglodytes*	O	
Passeriformes	Prunellidae	*Prunella collaris*		O
Passeriformes	Prunellidae	*Prunella montanella*		O
Passeriformes	Turdidae	*Turdus eunomus*		O
Passeriformes	Turdidae	*Turdus atrogularis*		O
Passeriformes	Turdidae	*Turdus cardis*		O
Passeriformes	Turdidae	*Monticola gularis*		O
Passeriformes	Turdidae	*Turdus naumanni naumanni*		O
Passeriformes	Turdidae	*Turdus mandarinus*		O
Passeriformes	Turdidae	*Turdus hortulorum*		O
Passeriformes	Turdidae	*Zoothera dauma*		O
Passeriformes	Turdidae	*Turdus obscurus*		O
Passeriformes	Turdidae	*Zoothera sibirica*		O

Passeriformes	Turdidae	*Turdus chrysolaus*		O
Passeriformes	Turdidae	*Turdus pallidus*		O
Passeriformes	Panuridae	*Paradoxornis webbianus*	O	
Passeriformes	Sylviidae	*Phylloscopus schwarzi*		O
Passeriformes	Sylviidae	*Phylloscopus inornatus*		O
Passeriformes	Sylviidae	*Phylloscopus proregulus*		O
Passeriformes	Sylviidae	*Phylloscopus tenellipes*		O
Passeriformes	Sylviidae	*Phylloscopus plumbeitarsus*		O
Passeriformes	Sylviidae	*Phylloscopus borealoides*		O
Passeriformes	Sylviidae	*Phylloscopus coronatus*		O
Passeriformes	Sylviidae	*Regulus regulus*		O
Passeriformes	Sylviidae	*Phylloscopus borealis*		O
Passeriformes	Sylviidae	*Phylloscopus fuscatus*		O
Passeriformes	Sylviidae	*Phylloscopus xanthodryas*		O
Passeriformes	Sylviidae	*Urosphena squameiceps*		O
Passeriformes	Sylviidae	*Locustella certhiola*		O
Passeriformes	Sylviidae	*Cettia diphone*		O
Passeriformes	Sylviidae	*Acrocephalus bistrigiceps*		O
Passeriformes	Sylviidae	*Locustella lanceolata*		O
Passeriformes	Muscicapidae	*Ficedula mugimaki*		O
Passeriformes	Muscicapidae	*Phoenicurus aursoreus*		O
Passeriformes	Muscicapidae	*Saxicola ferreus*		O
Passeriformes	Muscicapidae	*Saxicola maura*		O
Passeriformes	Muscicapidae	*Oenanthe pleschanka*		O
Passeriformes	Muscicapidae	*Muscicapa sibirica*		O
Passeriformes	Muscicapidae	*Muscicapa dauurica*		O
Passeriformes	Muscicapidae	*Luscinia cyane*		O
Passeriformes	Muscicapidae	*Luscinia sibilans*		O
Passeriformes	Muscicapidae	*Tarsiger cyanurus*		O
Passeriformes	Muscicapidae	*Luscinia calliope*		O
Passeriformes	Muscicapidae	*Cyanoptila cyanomelana*		O
Passeriformes	Muscicapidae	*Ficedula albicilla*		O
Passeriformes	Muscicapidae	*Luscinia svecica*		O
Passeriformes	Muscicapidae	*Ficedula zanthopygia*		O
Passeriformes	Muscicapidae	*Luscinia akahige*		O
Passeriformes	Muscicapidae	*Muscicapa dauurica*		O
Passeriformes	Monarchidae	*Terpsiphone atrocaudata*		O
Passeriformes	Aegithalidae	*Aegithalos caudatus*	O	
Passeriformes	Aegithalidae	*Aegithalos magnus*		O
Passeriformes	Remizidae	*Panurus biarmicus*		O
Passeriformes	Paridae	*Parus varius*	O	
Passeriformes	Paridae	*Parus venustulus*		O
Passeriformes	Paridae	*Parus major*	O	
Passeriformes	Paridae	*Parus monanus*	O	
Passeriformes	Paridae	*Parus palustris*	O	
Passeriformes	Paridae	*Parus ater*	O	
Passeriformes	Sittidae	*Sitta europacea*	O	
Passeriformes	Sittidae	*Sitta villosa*		O
Passeriformes	Certhiidae	*Certhia familiaris*		O
Passeriformes	Zosteropidae	*Zosterops japonicus*	O	
Passeriformes	Zosteropidae	*Zosterops erythropleurus*		O
Passeriformes	Emberizidae	*Emberiza variabilis*		O

Passeriformes	Emberizidae	*Emberiza chrysophrys*		O
Passeriformes	Emberizidae	*Emberiza elegans*	O	
Passeriformes	Emberizidae	*Emberiza cioides*	O	
Passeriformes	Emberizidae	*Emberiza fucata*		O
Passeriformes	Emberizidae	*Emberiza aureola*		O
Passeriformes	Emberizidae	*Emberiza rutila*		O
Passeriformes	Emberizidae	*Emberiza sulphurata*		O
Passeriformes	Emberizidae	*Emberiza bruniceps*		O
Passeriformes	Emberizidae	*Emberiza schoeniclus*		O
Passeriformes	Emberizidae	*Emberiza pusilla*		O
Passeriformes	Emberizidae	*Emberiza rustica*		O
Passeriformes	Emberizidae	*Emberiza tristrami*		O
Passeriformes	Emberizidae	*Calcarius lapponicus*		O
Passeriformes	Emberizidae	*Emberiza pallasi*		O
Passeriformes	Emberizidae	*Emberiza yessoensis*		O
Passeriformes	Emberizidae	*Emberiza spodocephala*		O
Passeriformes	Fringillidae	*Fringilla montifringilla*		O
Passeriformes	Fringillidae	*Carduelis spinus*		O
Passeriformes	Fringillidae	*Leucosticte arctoa*		O
Passeriformes	Fringillidae	*Pyrrhula pyrrhula*		O
Passeriformes	Fringillidae	*Eophona migratoria*		O
Passeriformes	Fringillidae	*Carduelis sinica*	O	
Passeriformes	Fringillidae	*Uragus sibiricus*		O
Passeriformes	Fringillidae	*Loxia curvirostra*		O
Passeriformes	Fringillidae	*Carpodacus roseus*		O
Passeriformes	Fringillidae	*Coccothraustes coccothraustes*		O
Passeriformes	Fringillidae	*Eophona personata*		O
Passeriformes	Fringillidae	*Carpodacus erythrinus*		O
Passeriformes	Fringillidae	*Carduelis flammea*		O
Passeriformes	Ploceidae	*Passer montanus*	O	
Passeriformes	Sturnidae	*Sturnus sericeus*		O
Passeriformes	Sturnidae	*Sturnus sturnius*		O
Passeriformes	Sturnidae	*Sturnus sinensis*		O
Passeriformes	Sturnidae	*Sturnus cineraceus*		O
Passeriformes	Sturnidae	*Sturnus vulgaris*		O
Passeriformes	Oriolidae	*Oriolus chinensis*		O
Passeriformes	Dicruridae	*Dicrurus macrocercus*		O
Passeriformes	Dicruridae	*Dicrurus hottentottus*		O
Passeriformes	Corvidae	*Cyanopica cyana*	O	
Passeriformes	Corvidae	*Pica pica*	O	
Passeriformes	Corvidae	*Corvus dauuricus*		O
Passeriformes	Corvidae	*Corvus corone*	O	
Passeriformes	Corvidae	*Pyrrhocorax pyrrhocorax*		O
Passeriformes	Corvidae	*Corvus macrorhynchos*	O	
Passeriformes	Corvidae	*Garrulus glandarius*	O	
