# Supplementary material for: Testing the causal mechanism of the peninsular effect in passerine birds from South Korea
Source: PLoS One. 2021 Jan 29;16(1):e0245958. doi: 10.1371/journal.pone.0245958 (PMC7846002; doi:10.1371/journal.pone.0245958)
Supplement: S2 Table — (PDF) [file pone.0245958.s002.pdf]

Independent variable	Scales (degree)	Number of cells	Curve patterns	Predictor	R^2	F	*P*
Latitude	0.125 × 0.125	589	Linear	Positive	0.0366	22.079	<0.001
			Quadratic		0.0376	11.451	<0.001
