# Supplementary material for: Testing the causal mechanism of the peninsular effect in passerine birds from South Korea
Source: PLoS One. 2021 Jan 29;16(1):e0245958. doi: 10.1371/journal.pone.0245958 (PMC7846002; doi:10.1371/journal.pone.0245958)
Supplement: S3 Table — Significant paths (P<0.05) are indicated in bold. (PDF) [file pone.0245958.s003.pdf]

Processes	Predictor variable	Response variable	Standard Estimate	Critical value	*P*-value	Beta
Recent deterministic	Latitude	Mean temperature	-0.66	-21.35	**<0.001**	**-1.27**
	Mean temperature	Passerine species richness	-0.28	-4.01	**<0.001**	**-0.76**
	Mean temperature	Habitat diversity	0.62	8.64	**<0.001**	**0.13**
	Mean temperature	Forest area	-1.15	-18.97	**<0.001**	**1.08**
	Mean temperature	LAI	-0.98	-14.76	**<0.001**	**0.01**
	Habitat diversity	Passerine species richness	0.14	3.19	**0.001**	**1.88**
	Forest area	Passerine species richness	0.33	3.29	**0.001**	**0.05**
	LAI	Passerine species richness	-0.04	-0.44	0.657	-0.58
Anthropogenic	Latitude	No. of patches	-0.36	-9.39	**<0.001**	**-48.89**
	No. of patches	Passerine species richness	0.01	0.29	0.773	0.00
Stochastic	LAI	Ratio of migrant species richness	0.03	0.34	0.730	0.01
	Forest area	Ratio of migrant species richness	-0.07	-0.64	0.525	0.00
	Habitat diversity	Ratio of migrant species richness	-0.58	-1.21	0.226	-0.01
	Mean temperature	Ratio of migrant species richness	-0.17	-2.33	**0.020**	**-0.01**
	Ratio of migrant species richness	Passerine species richness	0.53	16.02	**<0.001**	**34.26**
