# Supplementary material for: Testing the causal mechanism of the peninsular effect in passerine birds from South Korea
Source: PLoS One. 2021 Jan 29;16(1):e0245958. doi: 10.1371/journal.pone.0245958 (PMC7846002; doi:10.1371/journal.pone.0245958)
Supplement: Attachment — Submitted filename: Response to reviewers.docx [file pone.0245958.s005.docx]

PLOS ONE

Journal requirements:

When submitting your revision, we need you to address these additional requirements.

1. Please ensure that your manuscript meets PLOS ONE's style requirements, including those for file naming. The PLOS ONE style templates can be found at

<https://journals.plos.org/plosone/s/file?id=wjVg/PLOSOne_formatting_sample_main_body.pdf> and

<https://journals.plos.org/plosone/s/file?id=ba62/PLOSOne_formatting_sample_title_authors_affiliations.pdf>

2. We note that you have indicated that data from this study are available upon request. PLOS only allows data to be available upon request if there are legal or ethical restrictions on sharing data publicly. For more information on unacceptable data access restrictions, please see <http://journals.plos.org/plosone/s/data-availability#loc-unacceptable-data-access-restrictions>.

In your revised cover letter, please address the following prompts:

a) If there are ethical or legal restrictions on sharing a de-identified data set, please explain them in detail (e.g., data contain potentially sensitive information, data are owned by a third-party organization, etc.) and who has imposed them (e.g., an ethics committee). Please also provide contact information for a data access committee, ethics committee, or other institutional body to which data requests may be sent.

b) If there are no restrictions, please upload the minimal anonymized data set necessary to replicate your study findings as either Supporting Information files or to a stable, public repository and provide us with the relevant URLs, DOIs, or accession numbers. For a list of acceptable repositories, please see <http://journals.plos.org/plosone/s/data-availability#loc-recommended-repositories>.

We will update your Data Availability statement on your behalf to reflect the information you provide.

**▶ Thanks you for your contribution to this journal as an editor. We’ve checked the manuscript formats and added sentences explaining ethical and legal things in our cover letter.**

3. We note that Figures 2, 3, 4 in your submission contain map images which may be copyrighted. All PLOS content is published under the Creative Commons Attribution License (CC BY 4.0), which means that the manuscript, images, and Supporting Information files will be freely available online, and any third party is permitted to access, download, copy, distribute, and use these materials in any way, even commercially, with proper attribution. For these reasons, we cannot publish previously copyrighted maps or satellite images created using proprietary data, such as Google software (Google Maps, Street View, and Earth). For more information, see our copyright guidelines: <http://journals.plos.org/plosone/s/licenses-and-copyright>.

We require you to either (1) present written permission from the copyright holder to publish these figures specifically under the CC BY 4.0 license, or (2) remove the figures from your submission:

3.1.    You may seek permission from the original copyright holder of Figures 2, 3, 4 to publish the content specifically under the CC BY 4.0 license.

We recommend that you contact the original copyright holder with the Content Permission Form (<http://journals.plos.org/plosone/s/file?id=7c09/content-permission-form.pdf>) and the following text:

“I request permission for the open-access journal PLOS ONE to publish XXX under the Creative Commons Attribution License (CCAL) CC BY 4.0 (<http://creativecommons.org/licenses/by/4.0/>). Please be aware that this license allows unrestricted use and distribution, even commercially, by third parties. Please reply and provide explicit written permission to publish XXX under a CC BY license and complete the attached form.”

Please upload the completed Content Permission Form or other proof of granted permissions as an "Other" file with your submission.

In the figure caption of the copyrighted figure, please include the following text: “Reprinted from [ref] under a CC BY license, with permission from [name of publisher], original copyright [original copyright year].”

3.2.    If you are unable to obtain permission from the original copyright holder to publish these figures under the CC BY 4.0 license or if the copyright holder’s requirements are incompatible with the CC BY 4.0 license, please either i) remove the figure or ii) supply a replacement figure that complies with the CC BY 4.0 license. Please check copyright information on all replacement figures and update the figure caption with source information. If applicable, please specify in the figure caption text when a figure is similar but not identical to the original image and is therefore for illustrative purposes only.

The following resources for replacing copyrighted map figures may be helpful:

USGS National Map Viewer (public domain): <http://viewer.nationalmap.gov/viewer/>

The Gateway to Astronaut Photography of Earth (public domain): <http://eol.jsc.nasa.gov/sseop/clickmap/>

Maps at the CIA (public domain): <https://www.cia.gov/library/publications/the-world-factbook/index.html> and <https://www.cia.gov/library/publications/cia-maps-publications/index.html>

NASA Earth Observatory (public domain): <http://earthobservatory.nasa.gov/>

Landsat: <http://landsat.visibleearth.nasa.gov/>

USGS EROS (Earth Resources Observatory and Science (EROS) Center) (public domain): [http://eros.usgs.gov/#](http://eros.usgs.gov/)

Natural Earth (public domain): <http://www.naturalearthdata.com/>

**▶ All of these figures (2,3,4) were the ones that we drew by ourselves using the original source data. For example, we obtained bird observation data from the data provider** (website: <http://ecobank.nie.re.kr>), and then we drew the bird richness map (fig 4). Survey region map(fig2) and land cover map(fig3) were also drawn by ourselves.

[Note: HTML markup is below. Please do not edit.]

Reviewers' comments:

Reviewer's Responses to Questions

**Comments to the Author**

1. Is the manuscript technically sound, and do the data support the conclusions?

The manuscript must describe a technically sound piece of scientific research with data that supports the conclusions. Experiments must have been conducted rigorously, with appropriate controls, replication, and sample sizes. The conclusions must be drawn appropriately based on the data presented.

Reviewer #1: Yes

Reviewer #2: Yes

2. Has the statistical analysis been performed appropriately and rigorously?

Reviewer #1: Yes

Reviewer #2: Yes

3. Have the authors made all data underlying the findings in their manuscript fully available?

The [PLOS Data policy](http://www.plosone.org/static/policies.action#sharing) requires authors to make all data underlying the findings described in their manuscript fully available without restriction, with rare exception (please refer to the Data Availability Statement in the manuscript PDF file). The data should be provided as part of the manuscript or its supporting information, or deposited to a public repository. For example, in addition to summary statistics, the data points behind means, medians and variance measures should be available. If there are restrictions on publicly sharing data—e.g. participant privacy or use of data from a third party—those must be specified.

Reviewer #1: No

Reviewer #2: Yes

**▶ We made and added more information of our results in supplementary 2 and 3 files. And our raw data was uploaded when we upload revision files (raw data, excel file).**

4. Is the manuscript presented in an intelligible fashion and written in standard English?

PLOS ONE does not copyedit accepted manuscripts, so the language in submitted articles must be clear, correct, and unambiguous. Any typographical or grammatical errors should be corrected at revision, so please note any specific errors here.

Reviewer #1: Yes

Reviewer #2: Yes

5. Review Comments to the Author

Please use the space provided to explain your answers to the questions above. You may also include additional comments for the author, including concerns about dual publication, research ethics, or publication ethics. (Please upload your review as an attachment if it exceeds 20,000 characters)

Reviewer #1: The data could be uploaded in a table form per cell in a depository, the referred site is in Korean and it is hard for us to see. The results need more explicit description in the text to be able to follow the findings.

**▶ Thanks for your contribution to this paper for reviewer. More information about results of fig.4 and 5 were added in supplementary 2 and 3 files. These results were uploaded in a table form as you recommend (Table S2 and S3). And our raw data was uploaded when we upload revision files (raw data, excel file). And we will ask to make it possible in English for chief of the department proving the web site. Also, we modified parts of abstract and conclusion to make the smooth flow between results and conclusion (line no. 28~44, 291~309).**

Reviewer #2: Kim et al. peninsular effect

I like this paper very much. The peninsular effect is a fascinating one because it tells so much about the causes of species richness. When those peninsulas face southwards (in the northern hemisphere) then the declining species richness towards the tip runs counter to the large scale patterns of increasing diversity at lower latitudes. This is what this paper finds. Of course, one has to factor out the potentially confounding factors.
**▶ Thanks for your contribution to this paper for reviewer. We highlighted our finding in abstract and conclusion section (line no. 38~40, 304~306) as you recommended.**

I think the authors do that very well. I particularly enjoyed their figures that so clearly articulate the different hypotheses that they compare.

We understand that we will not get surveys from the northern part of the peninsula — for “political reasons” as the authors put it. Having been able to look southwards into North Korea from China (and across the border from Russia), I wonder if they authors might look at the diversity in China’s Amur tiger Amur leopard national park for a comparison of its richness. Which species present there are missing from South Korea? Doing this is not a criterion for publication, incidentally, just something the authors might consider.

**▶ As far as we know, Amur tiger and Amur leopard were presumed extinct in the Korean peninsula in early 20^th^ century. We asked a researcher who got interesting in amur tiger and leopard. She said that there is no any record about distribution data in South Korea.**

One minor quibble

The single model of passerine species richness showed the increasing pattern of species richness with increasing latitude in South Korea. Mean temperature induced by latitude gradient negatively affected LAI and forest area, and positively habitat diversity. However, passerine species richness was only influenced by mean temperature and forest area.
Expressions such as “negatively affected” are hard to understand. So, too, are using acronyms, such as LAI. A better way to write this paragraph would be

The single model of passerine species richness showed species richness increased with latitude in South Korea. Mean temperature increased with decreasing latitude, as did habitat diversity but leaf area index and forest area decline. However, mean temperature and forest area only influenced passerine species richness.
Comparable examples occur throughout.

**▶ Thank you so much for your considerate review. We remedied paragraph in abstract and conclusion section (line no. 28~44, 291~309), as you mentioned above. And we refrained using acronyms such as LAI in abstract and conclusion section. And for a detailed understanding of Fig.4 and 5, supplementary 2 and 3 files have been added.**

6. PLOS authors have the option to publish the peer review history of their article ([what does this mean?](https://journals.plos.org/plosone/s/editorial-and-peer-review-process#loc-peer-review-history)). If published, this will include your full peer review and any attached files.


If you choose “no”, your identity will remain anonymous but your review may still be made public.


**Do you want your identity to be public for this peer review?** For information about this choice, including consent withdrawal, please see our [Privacy Policy](https://www.plos.org/privacy-policy).

Reviewer #1: No

Reviewer #2: No

[NOTE: If reviewer comments were submitted as an attachment file, they will be attached to this email and accessible via the submission site. Please log into your account, locate the manuscript record, and check for the action link "View Attachments". If this link does not appear, there are no attachment files.]

While revising your submission, please upload your figure files to the Preflight Analysis and Conversion Engine (PACE) digital diagnostic tool, <https://pacev2.apexcovantage.com/>. PACE helps ensure that figures meet PLOS requirements. To use PACE, you must first register as a user. Registration is free. Then, login and navigate to the UPLOAD tab, where you will find detailed instructions on how to use the tool. If you encounter any issues or have any questions when using PACE, please email PLOS at [figures@plos.org](mailto:figures@plos.org). Please note that Supporting Information files do not need this step.
